# Tetanus toxin fragments and Bcl-2 fusion proteins: cytoprotection and retrograde axonal migration

**DOI:** 10.1186/s12896-018-0452-z

**Published:** 2018-06-11

**Authors:** Yasuhiro Watanabe, Takashi Matsuba, Mami Nakanishi, Mio Une, Ritsuko Hanajima, Kenji Nakashima

**Affiliations:** 1 Division of Neurology, Department of Brain and Neurosciences, Faculty of Medicine, Tottori University, Nishi-cho 36-1, Yonago, 683-8504 Japan; 2 Division of Bacteriology, Department of Microbiology and immunology, Faculty of Medicine, Tottori University, Nishi-cho 86, Yonago, 683-8503 Japan

**Keywords:** Apoptosis, Bcl-2, TeNT, TTC, Fusion protein, Neuron

## Abstract

**Background:**

Tetanus neurotoxin (TeNT) is taken up at nerve terminals and undergoes retrograde migration. The toxic properties of TeNT reside in the toxin light chain (L), but like complete TeNT, the TeNT heavy chain (TTH) and the C-terminal domain (TTC) alone can bind and enter into neurons. Here, we explored whether atoxic fragments of TeNT could act as drug delivery vehicles in neurons. In this study, we used Bcl-2, a protein known to have anti-apoptotic properties in vivo and in vitro, as a parcel to couple to TeNT fragments.

**Results:**

We expressed Bcl-2 and the TTC fragments alone, and also attempted to express fusion proteins with the Bcl-2 coupled at the N-terminus of TTH (Bcl2-TTH) and the N- and C-terminus of TTC (TTC-Bcl2 and Bcl2-TTC) in mammalian (Cos7 cells) and *Escherichia coli* systems. TTC and Bcl-2 were efficiently expressed in *E. coli* and Cos7 cells, respectively, but Bcl-2 and the fusion proteins did not express well in *E. coli.* The fusion proteins were also not expressed in Cos7 cells. To improve the yield and purity of the fusion protein, we genetically deleted the N-terminal half of TTC from the Bcl2-TTC fusion to yield Bcl2-*h*TTC. Purified Bcl2-*h*TTC exhibited neuronal binding and prevented cell death of neuronal PC12 cells induced by serum and NGF deprivation, as evidenced by the inhibition of cytochrome C release from the mitochondria. For in vivo assays, Bcl2-*h*TTC was injected into the tongues of mice and was seen to selectively migrate to hypoglossal nuclei mouse brain stems via retrograde axonal transport.

**Conclusions:**

These results indicate that Bcl2-*h*TTC retains both Bcl-2 and TTC functions and therefore could be a potent therapeutic agent for various neurological conditions.

## Background

**Fig. 1:**
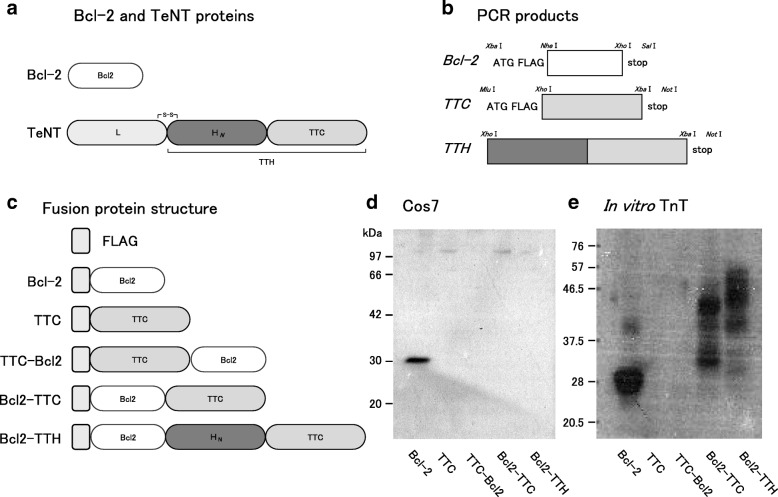
**a** Schematic representation of Bcl-2 and TeNT proteins. The active TeNT is composed of a light chain zinc protease (L, 50 kDa) and a heavy chain (TTH, 100 kDa) that is linked to the L chain during translation and requires activation and reduction of disulfides to dissociate. The TTH governs neuronal cell binding, uptake, and transport. The TTH chain is composed of two domains: an N-terminal 407-amino-acid domain (H_*N*_, 45 kDa) and a C-terminal 452-amino-acid domain (TTC, 55 kDa). The TTC domain alone retains neuronal binding and migration abilities. **b** PCR procedure. The cDNAs for Bcl-2, TTC and TTH were cloned using primers designed to introduce a start codon (ATG) or stop codon, FLAG sequence or restriction enzyme sites (Table 1). The selected restriction enzymes share compatible cohesive ends, e.g., *Xba*I and *Nhe*I, to facilitate construction of fusion protein cDNA. **c** Fusion proteins. Along with the Bcl-2 protein and the TTC fragment alone, TTC-Bcl2 and Bcl2-TTC fusion proteins were produced with the Bcl-2 fused at the C-terminus and N-terminus of TTC, respectively. A Bcl2-TTH fusion was also generated. Each fusion protein contained an N-terminal FLAG sequence. The estimated molecular weights were: Bcl-2: 26 kDa, TTC: 55 kDa, TTC-Bcl2: 81 kDa, Bcl2-TTC: 81 kDa, Bcl2-TTH: 126 kDa. **d** Fusion proteins expressed in Cos7 cells. Fusion proteins were detected with anti-FLAG antibody. Only Bcl-2 expression was confirmed. **e** In vitro transcription/translation (TnT). During in vitro expression, Bcl-2 was expressed with an appropriate molecular size, whereas Bcl2-TTC and Bcl2-TTH were smaller than expected, likely due to premature termination of protein synthesis. When the TTC fragment was located at the N-terminus of the fusion proteins (e.g. TTC and TTC-Bcl2), no protein expression was detected

The blood brain barrier (BBB) protects the central nervous system (CNS) from harmful substances circulating in the blood stream [1]. However, the BBB poses a significant obstacle for drug delivery to the CNS [2]. Tetanus neurotoxin (TeNT), produced by *Clostridium tetani*, is one of the most potent neurotoxins in humans. Following uptake at neuromuscular junctions, the TeNT travels along motor axons via retrograde axonal transport and then enters the CNS where it blocks release of inhibitory neurotransmitters from spinal interneurons [3]. TeNT causes tetanus, which is characterized by painful muscular contractions and spasms as well as seizure; in severe cases TeNT exposure can be fatal. TeNT is composed of a light chain (L) and a heavy chain (TTH) (Fig. 1a) [3]. The C-terminal domain half of TTH (TTC) alone can target neuronal cells and undergo retrograde axonal transport [4, 5]. Furthermore, several reports indicate that the C-terminus may have an antiapoptotic effect on neurons [5–7]**.** Both in vitro and in vivo studies have shown that the TTC induces neuroprotection [5, 8, 9].

Based on these properties, we considered that TTC could be a promising vehicle to deliver drug cargos to neurons. To explore this possibility, we engineered fusion proteins containing various TeNT fragments. We chose B-cell leukemia/lymphoma 2 protein (Bcl-2) [10] as a partner protein, because Bcl-2 is one of the most potent anti-apoptotic proteins [10] and has an appropriate size (26 kDa) to act as a fusion partner. We tested these fusion proteins in protein expression systems or using purified fusion proteins to determine whether the fusion products retained both anti-apoptotic and neuronal migration properties.

## Methods

### Culture of *C. tetani*

*C. tetani* strain KZ1174, corresponding to Harvard A-47, was obtained from the Department of Bacteriology, School of Medicine, Kanazawa University, and was handled according to the Manual for Biosafety Relating to Pathogenic Bacteria (Japanese Society for Bacteriology). The bacteria were anaerobically grown overnight at 37 °C in 2 ml cooked meat medium (Difco, USA). The cells were subsequently collected by centrifugation, and plasmid DNA was extracted using the Wizard Plus DNA purification system (Promega, USA).

### Plasmid construction

**Fig. 2:**
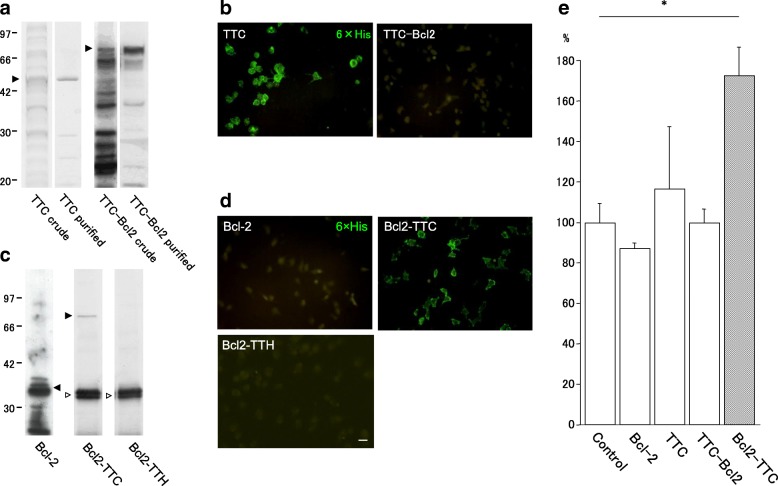
**a** Expression and purification of N-terminal TTC fusion proteins in prokaryotes. Each cDNA coding TTC or TTC-Bcl2 was introduced into a prokaryotic expression vector with an N-terminal 6 × His tag sequence (His- His- His- His- His- His). CBB staining was used for detection of both crude and purified TTC. TTC-Bcl2 fusion proteins were further detected with anti-Bcl-2 antibody (arrowhead). **b** Cell surface binding of fusion proteins. Binding of approximately 40 nM (as estimated from SDS-PAGE) TTC or TTC-Bcl2 to NGF-differentiated PC12 cells immuno-detected with an anti-His tag antibody. Purified TTC bound to the cell surface of neuronal-differentiated PC12 cells whereas TTC-Bcl2 fusion protein did not. The results suggest that binding to neuronal cells could require a free TTC C-terminus. Scale bar = 25 μm. **c** Expression and purification of N-terminal TTC fusion proteins in prokaryotes. Bcl2-TTC and Bcl2-TTH fusion proteins, as well as Bcl-2 alone, tagged with 6 × His tag sequence at the N-terminus were expressed in *E. coli*. Fusion proteins detected with an anti-Bcl-2 antibody (arrowhead). For Bcl2-TTC and Bcl2-TTH, preparations of purified fusion proteins contained smaller-sized fragments (30 kDa) that likely represent C-terminal truncated proteins. **d** Cell surface binding of fusion proteins. Binding of approximately 40 nM (as estimated from SDS-PAGE) of fusion proteins to NGF-differentiated PC12 cells immuno-detected with an anti-His tag antibody. Purified Bcl2-TTC bound to the cell surface of neuronal-differentiated PC12 cells, whereas Bcl-2 and Bcl2-TTH fusion proteins did not. Scale bar = 25 μm. **e** Cell viability assays. PC12 cells were differentiated by NGF and then exposed to each of the recombinant proteins for 1 h. After exposure, serum and NGF were withdrawn. PC12 cell survival was evaluated 48 h after exposure. Compared to control only, Bcl2-TTC significantly increased PC12 cell survival following serum and NGF withdrawal (*: *p* < 0.05). Values are means ± s.e. (*n* = 8)

Plasmid pSK Bcl-2 containing human Bcl-2 cDNA was kindly provided from Prof. Y. Tsujimoto (Department of Medical Genetics, Osaka University Medical School, Japan). The coding regions for TTC, TTH and Bcl-2 were amplified by PCR using mutagenic primers (Table 1). Several restriction sites and epitope expression tag sequences were introduced in the primers (Fig. 1b) to facilitate subsequent cDNA ligation as well as protein detection procedures. The FLAG tag octapeptide sequence (Asp-Tyr-Lys-Asp-Asp-Asp-Asp-Lys) was introduced for eukaryotic protein expression. Various fusion proteins are designated as shown in Fig. 2a by genetically joining TTC with Bcl-2 using compatible cohesive sites. The designations indicate the order of the fusion from N- to C-terminus. For example, Bcl2-TTC, in which TTC was fused to the C-terminus of Bcl-2, was produced by ligating the *Xho*I sites of Bcl-2 and TTC, whereas TTC-Bcl2 was produced by ligating the TTC *Xba*I site to the Bcl-2 *Nhe*I site. Bcl2-TTH, which contains the intact TeNT heavy chain, was produced in a similar manner.

**Table 1:** Primer sequences used to produce fusion proteins

Primer name	Sequence	Modified sequence(s)
Bcl2 sens	5’-GATCTAGAATGGACTACAAAGACGACGACGACAAGGCTAGCATGGCGCACGCTGGGAGA-3′	*Xba*I, start codon, FLAG, *Nhe*I
Bcl-2 rev	5’-GGGTCGACTTACTCGAGAATCTTGTGGCCCAGATAGGC-3’	*Xho*I, stop codon, *Sal*I
TTC sens	5’-AAACGCGTATGGACTACAAAGACGACGACGACAAGCTCGAGATGAAAAATCTGGATTGTT-3’	*Mlu*I, start codon, FLAG, *Xho*I
TTC rev	5’-CAGCGGCCGCTCATCTAGAAATATCATTTGTCCATCCTTC-3’	*Xba*I, stop codon, *Not*I
TTH sens	5’-AGACTCGAGTCATTAACAGATTTAGGAGGA-3’	*Xho*I
TTH rev	similar to TTC rev

### Expression of fusion proteins in Cos7 cells and in vitro transcription/translation (TnT) system

For eukaryotic expression, each construct was introduced into pCI vectors (Promega). The fusion proteins were expressed in Cos7 cells using a calcium phosphate method [11] and rabbit reticulocyte lysates in an in vitro transcription/translation (TnT) system according to the manufacturer’s protocol (Promega).

### Expression and purification of recombinant his-tagged proteins

**Fig. 3:**
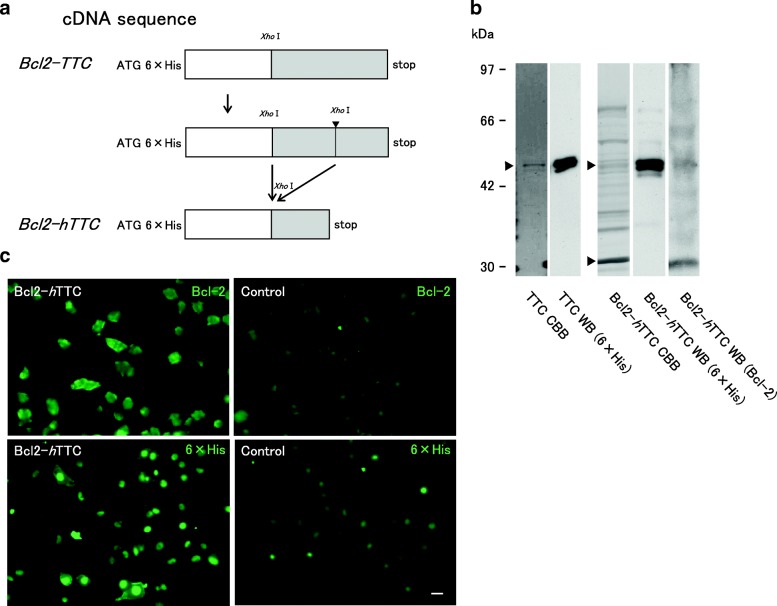
**a** Truncation of TTC fragment from Bcl2-TTC. The 254 N-terminal residues of TTC were genetically deleted by introducing an additional *Xho*I restriction site, such that the resulting Bcl2-*h*TTC fusion protein consisted of the 6 × His tag sequence, Bcl-2 and the C-terminal 204 residues of TTC. The estimated molecular weight of Bcl2-*h*TTC was 53 kDa, which is almost identical to that of full-length TTC (55 kDa). **b** Purification and detection of TTC and Bcl2-*h*TTC. Total protein (0.025 μg and 0.10 μg for TTC and Bcl2-*h*TTC, respectively) was loaded onto the gel. Purified fusion proteins were stained with CBB, and were further detected using an anti-6 × His antibody and anti-Bcl-2 antibody. **c** Cell surface binding of fusion proteins. Neuronal binding capacity of Bcl2-*h*TTC to differentiated PC12 cells as detected by an anti-Bcl-2 antibody and anti-6 × His antibody. Bcl2-*h*TTC showed neuronal binding activity that was similar to that of TTC (Fig. 2b) and Bcl2-TTC (Fig. 2d). Differentiated PC12 cells added vehicle alone were used as a control. Scale bar = 25 μm

**Fig. 4:**
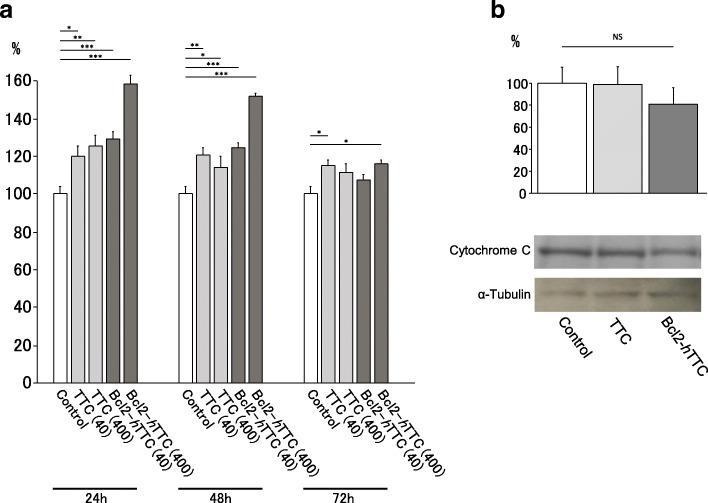
**a** Anti-apoptotic effect of fusion proteins in serum and growth factor-deprived PC12 cells. NGF-differentiated PC12 cells were NGF and serum deprived and then were exposed to various concentration of Bcl2-*h*TTC, TTC, or vehicle alone (controls). Cell survival was assessed by an MTT assay. The percentage of cell survival was determined 24, 48 and 72 h after exposure. An anti-apoptotic effect of Bcl2-*h*TTC, particularly at 400 nM, was observed compared to the control as analyzed by a post hoc test (Fisher’s PLSD). TTC alone also showed a mild cytoprotective effect. * *p* < 0.01, ** *p* < 0.001, *** *p* < 0.0001. Values were means ± s.e. (*n* = 8). **b** Cytosolic distribution of cytochrome C in PC12 cells following withdrawal of trophic support. In PC12 cells pretreated with Bcl2-*h*TTC (*n* = 4), the amount of cytochrome C release was less than that seen for untreated PC12 cells (control, *n* = 4) and cells treated with TTC (*n* = 4). Although these did not reach statistical significance (*p* = 0.39, control vs. Bcl2-*h*TTC)

For prokaryotic expression, the entire coding regions, except for the FLAG sequence, were transferred from the pCI-based vector to the pRSET C vector (Invitrogen, USA), which contains a 6 × histidine (His) tag sequence at the N-terminus to ease purification via immobilized metal affinity chromatography (IMAC) as well as to facilitate detection of fusion proteins. Briefly, we used upstream and downstream primers with the sequence 5’-ATGCATAAGCTTGACGACGACGACAAG-3′ and 5’-AAGCTTATGCATATCTTATCATGTCTGCTC-3′ (the underlined segments of both primers indicate *Hind*III sites), respectively, in PCR reactions with the pCI-based vector as a template. The PCR products were cloned into T-vectors, and the open reading frame of each construct was confirmed by sequencing. We then introduced the PCR products into the pRSET C vector digested with *Hind*III (Figs. 3a and 4a).

**Fig. 5:**
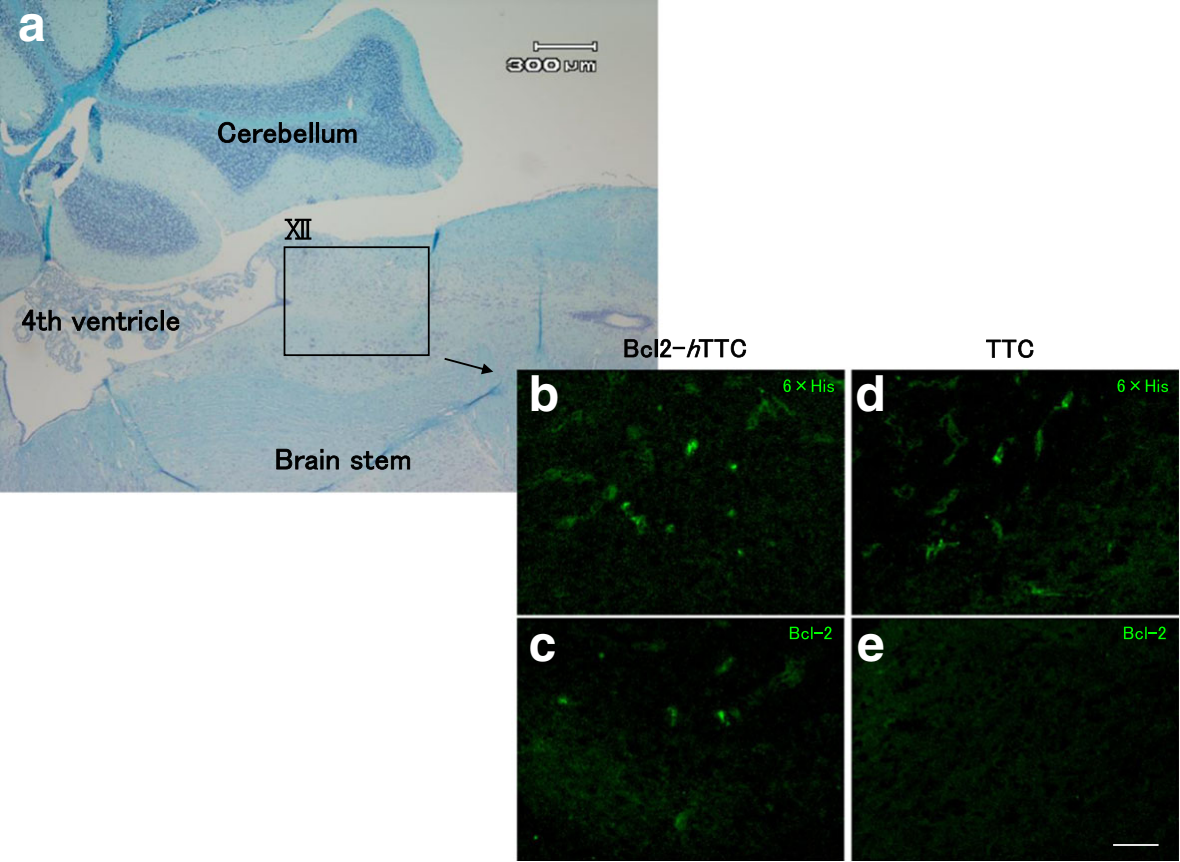
Immunohistochemical observations of brain stems from B6CJ mice injected with fusion proteins. Sagittal section of the brain stem (**a**: Klüver-Barrera staining) was immunohistochemically stained using anti 6 × His antibody (**b** and **d**) or anti-Bcl-2 antibody (**c** and **e**). Fusion proteins were injected into the tongue muscle of mice and were seen to accumulate and localize in hypoglossal neurons (XII). Bcl2-*h*TTC reacted with both 6 × His (**b**) and Bcl-2 (**d**) antibodies, whereas TTC only reacted with the anti-6 × His antibody (**c**). Scale bar = 100 μm

The C-terminal half of TTC (residues 1111–1315, hereafter termed *h*TTC) was previously shown to be sufficient for interaction with the neuronal cell surface [12]. As described by Herreros et al. [12], we deleted the N-terminal half of TTC (residues 856–1110) from Bcl2-TTC to produce Bcl2-*h*TTC (Fig. 5a) by introducing an additional *Xho*I restriction site around TTC residue 856 using the primer (*h*TTC forward): 5’-AGTCTCGAGTCTATAACCTTTTTAAG-3′ (the *Xho*I site is underlined) in a PCR reaction as described above.

The resulting vectors were used to transform *Escherichia coli* (*E. coli*) BL21(DE3)pLysS cells (Promega) cells, which were cultured overnight in 5 ml terrific broth (Gibco, USA). Then, 1 L terrific broth with carbenicillin (50 μg/ml) and chloramphenicol (35 μg/ml) was inoculated with overnight cultures of *E. coli* transformed with the various constructs. When the cultures reached OD_650_ of 0.8, production of fusion proteins was induced by the addition of isopropyl-β-D-thiogalactopyranoside (IPTG) to a final concentration of 0.5 mM. The cells were incubated at 30 °C for an additional 4 h and then collected. Purification of 6 × His-tagged proteins was performed by IMAC using cobalt-based resins (TALON, Clontech, USA). The His-tagged proteins were eluted from the resin with a buffer containing 250 mM imidazole and imidazole was removed by microdialysis using a Mini Dialysis kit with an 8 kDa cut-off (Amersham, UK) (Figs. 3b, 4b and 5b).

### Western blot analysis

The samples derived from *E. coli* were separated by SDS-PAGE and transferred onto Immobilon P PVDF membranes (Millipore, USA) or stained with Coomassie brilliant blue (CBB). The recombinant proteins were detected with anti-6 × His antibody (6-Histidine Ab-1, 1100, NeoMarkers, USA) or anti-Bcl-2 antibody (ΔC 21, Santa Cruz Biotechnology, USA), followed by horseradish peroxidase (HRP)-linked secondary antibodies and detection with the ECL detection system (Amersham).

### Binding experiment

The binding of the fusion proteins to PC12 cells was examined as described previously [13] with minor modifications. PC12 cells were maintained in Dulbecco’s modified minimum Eagle medium (DMEM) with 10% fetal bovine serum (FBS). For neuron-like differentiation, PC12 cells were plated in an 8-well chamber slide (Nalge Nunc, USA) at 1.0 × 10^3^ cells/well and differentiation was induced by treatment with 50 ng/ml 7S-nerve growth factor (7S-NGF, Wako, Japan) in DMEM with 10% FBS. After the cells were allowed to differentiate for 7 days, they were washed three times with Hanks’ balanced salt solution (HBSS) before 40 nM (as estimated from SDS-PAGE protein densities) of each fusion protein was added. The cells were incubated with the fusion protein for 1 h at 4 °C. After exposure, the cells were washed with HBSS three times, and then stained using anti-6 × His antibody without cell permeabilization. The cells were incubated for 1 h with anti-mouse IgG conjugated with fluorescein-isothiocyanate (FITC) as a secondary antibody and evaluated using fluorescent microscopy.

### Cell survival assay

A cell survival assay was performed according to a previous report [14]. Differentiated PC12 cells cultured in 96-well plates were washed three times with HBSS and then exposed to various concentrations of each fusion protein for 1 h. Following the exposure, the cells were again washed three times with HBSS and then maintained for 48 h in DMEM without NGF or FBS to induce cell death. Cell viability was determined by a colorimetric 3-(4,5-dimethylthiazol-2-yl)-2,5-diphenyltetrazolium bromide (MTT) metabolic activity assay in a microtiter plate reader at 570 nm.

Statistical analysis was performed by one-way ANOVA and post hoc test.

### Cytochrome C release

Bcl-2 is known to suppress apoptosis by inhibiting the release of cytochrome C from the mitochondria to the cytosol. Soluble cytoplasmic fractions of cell lysates were prepared according to a previous report [15]. Briefly, differentiated PC12 cells cultured in T-75 flasks were treated with Bcl2-*h*TTC, TTC, or with vehicle alone as a control. The cells were rinsed twice using ice-cold phosphate-buffered saline (PBS) and harvested after the addition of a small volume (100 μl) of cold sucrose-supplemented cell extract buffer {250 mM sucrose, 20 mM HEPES (pH 7.4), 10 mM KCl, 1.5 mM MgCl_2_, 1 mM EGTA, 1 mM EDTA, 1 mM dithiothreitol, and a protease inhibitor cocktail tablet (cOmplete mini; Roche, Switzerland)}. The pelleted cells were resuspended in the above buffer, incubated on ice for 30 min, homogenized, and centrifuged at 14,000 x g for 15 min. The supernatants comprising the cytoplasmic/soluble fraction were stored at − 80 °C before analysis. Total protein was quantified by the Bradford method. Lysates with equal amounts of protein were loaded onto SDS-PAGE and detected with a mouse anti-cytochrome C antibody (clone 6H2.B4, 1:100 dilution) (BioLegend, USA). Monoclonal anti-α-tubulin (clone DM1A, 1:500 dilution) (Sigma-Aldrich, USA) was used as an internal standard.

### Retrograde axonal migration in vivo

C57BL/6CR mice were purchased from Shimizu laboratory supplies (Japan). The animal experimental procedure was approved by the Tottori University animal experiment committee. Each fusion protein (10 μg TTC or Bcl2-*h*TTC) or PBS alone (10 μl per animal) was injected in the tongue muscle of 14 week-old mice using a Hamilton syringe under general anesthesia with pentobarbital (Somnopentil, Kyoritsu Pharmaceutical, Japan). After a 24-h post injection period to allow migration of the injected protein, the mice were deeply anesthetized with pentobarbital and transcardially perfused with saline, followed by 4% paraformaldehyde. Brains were removed and dehydrated in sucrose, then mounted in Tissue-Tek before sectioning into 8 μm-thick slices using a cryostat. Sagittal sections were stained with the Klüver-Barrera method or immunostained with anti-Bcl-2 or anti-6 × His antibody.

## Results

### Fusion protein expression in Cos7 cells and by in vitro TnT

Each expression construct was introduced into a mammalian expression vector. In Cos7 cells (Fig. 1d), Bcl-2 expression was detectable, but the expression levels of the fusion proteins were below the limits of detection. Bcl-2 was also readily detected in an in vitro TnT (Fig. 1e). Meanwhile, the molecular weights of both Bcl2-TTC and Bcl2-TTH were lower than expected, presumably due to premature termination of protein synthesis. When TTC was located at the N-terminus of the fusion proteins, i.e., both TTC and TTC-Bcl2, the proteins were hardly detectable.

These results are likely explained by the different protein origins in that native TeNT is produced by anaerobic bacteria whereas the mammalian protein Bcl-2 alone could be produced in eukaryotic expression systems involving either Cos7 or in vitro TnT.

### Expression, purification and binding assay of N-terminal-TTC or N-terminal-Bcl-2 fusion proteins

As the above experiment indicated that protein expression could be species-sensitive, we undertook bacterial expression assays with fusions having N-terminal TTC, i.e., TTC and TTC-Bcl2. Both were expressed in *E. coli* and successfully purified by IMAC (Fig. 2a, closed arrowheads). TTC is visible as a single band in CBB staining, whereas TTC-Bcl2 could only be detected by western blotting. Next, we evaluated the binding activity of fusion proteins (TTC and Bcl2-TTC) to differentiated neuronal PC12 cells. TTC displayed neural binding properties, but the TTC-Bcl2 fusion protein did not (Fig. 2b).

We then expressed fusion proteins with N-terminal Bcl-2, Bcl-2 alone, Bcl2-TTC and Bcl2-TTH. Only small amounts of Bcl2-TTC fusion protein were obtained, and for Bcl2-TTH, insufficient amounts of purified fusion protein for additional experiments were obtained, both due to premature truncations after translation of the Bcl-2 segment. An experiment to determine binding activity indicated that Bcl2-TTC, but not Bcl-2 alone, bound to PC12 cells (Fig. 2d). This outcome is in contrast to results for the C-terminal Bcl2 fusion protein, TTC-Bcl2 (Fig. 2b), which showed no binding to PC12 cells, indicating that TeNT fusion proteins must have a free C-terminus to exhibit binding to neuronal cells.

Differentiated PC12 cells were exposed to about 40 nM Bcl-2, TTC, TTC-Bcl2, Bcl2-TTC, or buffer alone (control) and then maintained for 48 h with DMEM without either NGF or FBS to induce cell death. TTC, TTC-Bcl2 and Bcl-2 showed no protective effect in our experimental condition. Only Bcl2-TTC partially prevented PC12-cell death compared to control (Fig. 2e).

### Short version of Bcl2-TTC (Bcl2-*h*TTC)

Our experiment indicated that as far as interactions with neurons were concerned, Bcl2-TTC retained TTC biological properties (Fig. 2d) and was cytoprotective (Fig. 2e). However, the yield and purity of the purified protein was not sufficient to proceed with further experiments. Meanwhile, *h*TTC (TTC residues 1111–1315) was previously shown to be sufficient for neuronal binding and interactions with neuronal cell surface [12]. Based on these findings we genetically introduced an additional *Xho*I site (Fig. 3a) to remove the N-terminal half of TTC using *Xho*I restriction sites framing the N-terminal region. After *E. coli* expression and IMAC purification, Bcl2-*h*TTC was successfully purified, although the overall purity of this fusion protein was lower than that obtained for TTC alone (Fig. 2b). Similar to Bcl2-TTC (Fig. 2d), Bcl2-*h*TTC showed neuronal binding activity (Fig. 3c).

### Cell survival assay

After PC12 cells were differentiated with NGF, serum and NGF were withdrawn from the culture medium and simultaneously various concentrations of TTC, Bcl2-*h*TTC, or buffer alone (control) were added to the medium. Addition of Bcl2-*h*TTC partially prevented PC12-cell death in a dose dependent manner. Compared to untreated control cells, the differences between two groups were statistically significant (Fig. 4a). TTC also showed slight cytoprotective properties, but this activity appeared to reach ceiling at lower concentration (40 nM) (Fig. 4a).

### Cytochrome C assay

The small heme-containing protein cytochrome C is released from mitochondria during the early stages of apoptosis. Bcl-2 can prevent cell death by blocking cytochrome C release from the mitochondria to the cytosol. We therefore assayed cytochrome C release in the cytosolic fraction of PC12 cells that had been induced to undergo apoptosis by serum and NGF withdrawal. The amount of cytochrome C release in PC12 cells treated with Bcl2-*h*TTC was lower than that seen for cells treated with TTC or without treatment, suggesting that the fusion protein reduced apoptosis (Fig. 4b).

### Retrograde axonal migration

To test the in vivo effects of TTC or Bcl2-*h*TTC, purified proteins were injected into the tongue muscle of mice. Knowing that Bcl-2 did not show neural binding (Fig. 2d) or cytoprotective activity (Fig. 2e) when added extracellularly, we did not test Bcl-2 alone in this experiment. At 24 h after the injection, the mice were sacrificed. During the time between injection and sacrifice, neither tetanus-like symptoms nor other neurological symptoms were noted. In animals injected with Bcl2-*h*TTC, isolated soma hypoglossal nuclei were immune-positive for anti-Bcl-2 antibody and 6 × His antibody, indicating that the fusion protein was transported across the BBB (Fig. 5b and c). Meanwhile, in mice that received TTC alone, only the cytoplasm of hypoglossal nuclei was positive for 6 × His (Fig. 5d and e), and no positive staining was seen in mice that received injection of PBS alone (data not shown).

## Discussion

### Bcl-2 is neuroprotective

Bcl-2 inhibits cell death in culture induced by various diverse stresses, including growth factor withdrawal, addition of calcium ionophore, glucose withdrawal, membrane peroxidation, free radical attack, and hypoxia [16]. Further, Bcl-2 overexpression in a mouse model of amyotrophic lateral sclerosis (ALS) delayed disease onset [17, 18] and prolonged survival [17]. Bcl-2 protein is a relatively small protein that could be produced and purified as a fusion protein. Here we explored whether the C-terminal portion of TeNT, TTC, could deliver a fused Bcl-2 protein into the intracellular space of neuronal cells to confer neuroprotection and in turn neuronal survival.

One proposed mechanism by which Bcl-2 inhibits apoptosis involves binding to other Bcl-2 protein family members such as Bax and Bad. This binding could suppress pore formation in the mitochondrial outer membrane and prevent release of pro-apoptotic proteins, e.g., cytochrome C, to the cytosol [19]. Bcl-2 has indirect antioxidant activity in response to elevated mitochondrial production of reactive oxygen species (ROS) [19].

### TTC also improves cell survival

In the cell survival experiment, we confirmed that Bcl2-*h*TTC showed an anti-apoptotic effect relative to control cells. However, we also noticed a slight, but distinct, protective effect of TTC in the treated group compared to the control group. This finding is consistent with those of several previous studies that implicated the TTC of TeNT in neurotrophic signaling pathways and anti-apoptotic processes [5, 20]. The neuroprotective properties of TTC could be manifested through the phosphatidylinositol-3 kinase (PI-3 K)/Akt signal pathway [20, 21] via the neurotrophin receptors p75 and tyrosine kinase receptor B (TrkB) [6]. Recent research also revealed that the binding properties of TTC to gangliosides GD1b and GT1b were needed for internalization of this protein by neurons [22].

This cytoprotective property of TTC is completely incompetent when TTC is incorporated as a functional part of TeNT. Fusing with Bcl-2, we successfully intensified the cytoprotective ability as well as dose dependent kinetics of TTC.

### TTC naturally accumulates into target

The BBB confers a privileged status on brain and spinal cord tissue to isolate and protect it from other regions and fluids of the body. As such, many molecules that exert neuroprotective abilities in vitro fail to show similar activity in vivo. For example, ciliary neurotrophic factor (CNTF) has a potent survival effect on motor neurons in vitro [23], but neither subcutaneous [24] nor intramuscular [25] injection of CNTF in human ALS patients produced motor improvement, and in fact promoted adverse systemic reactions [24, 25]. This result indicates that delivery of CNTF to the CNS was likely blocked by the BBB. Given its ability to transcytose between neurons, the TeNT C-terminal fragment TTC could be used as part of a fusion protein to overcome the BBB obstacle. Use of TTC to deliver molecular cargo is supported by the finding that TTC fusion proteins can naturally accumulate in neurons. This property would minimize the amount of fusion protein needed for injection and perhaps reduce negative side effects. Moreover, unlike virus vector mediated gene therapy, the use of TTC is free from risk of viral infection and oncogenicity.

Several studies have already successfully exploited the useful properties of TTC [26–28]. To treat GM2 gangliosidosis (Tay-Sachs and Sandhoff diseases), β-*N*-acetylhexosaminidase A (Hex A) was coupled to TTC by an in vitro disulfide linkage [26]. Following treatment of cultured cells from a feline model of GM2 gangliosidosis with TTC-Hex A, lysosomal accumulation of GM2 ganglioside was successfully eliminated [26]. In another case, the free-radical scavenging enzyme SOD1 was genetically fused to TTC and expressed in *E. coli* [27]. In a rat model of transient focal ischemia/reperfusion, the SOD1-TTC fusion protein significantly reduced brain infarct volume [28]. Neurotrophic factors such as IFG-1 [29] and GDNF [30] have also been coupled with TTC. Despite raising levels of exogenous IGF-1 in the spinal cord, intramuscular or intrathecal administration of IGF1-TTC had no significant effect on disease progression or survival of ALS model mice with high expression levels of mutant SOD1^G93A^ [29], whereas the genetic fusion of TTC with GDNF increased survival by 9 days and mitigated symptoms in ALS animal models [30]. These experiments clearly showed the possibility that TTC could be used as a universal carrier to deliver therapeutic agents either to the outer surface or cytosol of neurons.

Another promising strategy for use of TTC fusions could involve a naked plasmid technique wherein plasmids encoding TTC fusion constructs are injected into host cells to drive fusion protein expression [31, 32]. Such a strategy was successfully used for a fusion of TTC with green fluorescent protein (GFP) [31] or β-galactosidase [32]. However, under our experimental conditions, in Cos7 cells only Bcl-2 alone expressed to detectable levels and we could not express sufficient amounts of fusion proteins. Intriguingly, Carlton et al. reported that the fusion proteins between TTC and Bcl-xL, another anti-apoptotic Bcl-2 family protein, improved cell survival and decreased apoptosis in glutamate-mediated excitotoxicity of SH-SY5Y neuronal cells [33]. Consistent with our results, in that study in vitro internalization of Bcl-xL fusion protein required that the TTC be fused at the C-terminus of Bcl-xL [33]. Our experiment using mice hypoglossal nerves further indicated that a Bcl-2 fusion protein with C-terminal TTC fusion would work even in vivo.

## Conclusions

In conclusion, we showed in vitro that Bcl2-*h*TTC fusion protein reinforced the inherent cytoprotective effect of TTC. Our kinetic analysis of the fusion protein in mice in vivo successfully showed that both Bcl2-*h*TTC fusion protein and TTC alone underwent retrograde axonal migration from the site of injection in the tongue muscle to hypoglossal nuclei. After addressing several issues such as protein expression efficacy and final yield of the fusion protein, we expect to confirm the anti-apoptotic effect of the Bcl-2-TTC fusion protein in animal models of neurological diseases, such as motor neuron disease.

## Abbreviations

Bcl-2: B-cell leukemia/lymphoma 2
CNS: central nervous system
*h*TTC: carboxyl half of TTC
NGF: nerve growth factor
TeNT: tetanus neurotoxin
TTC: carboxyl half of TTH
TTH: TeNT heavy chain
